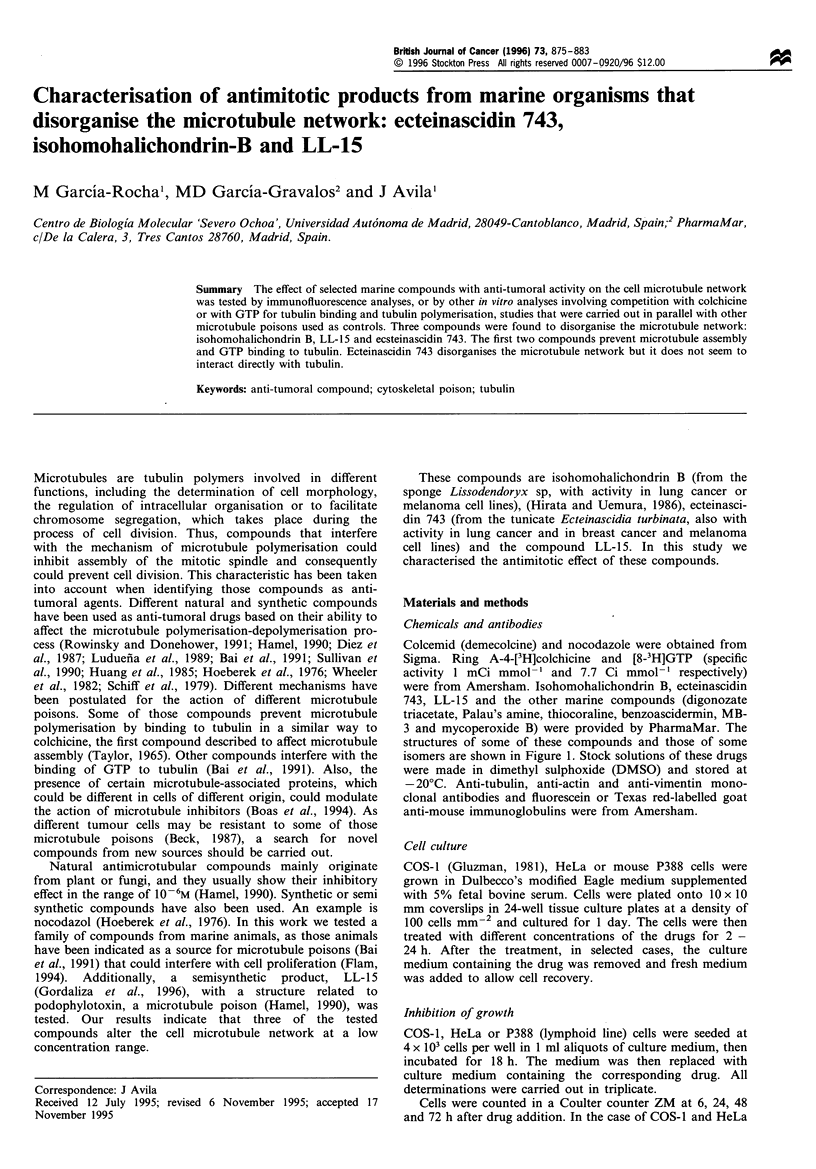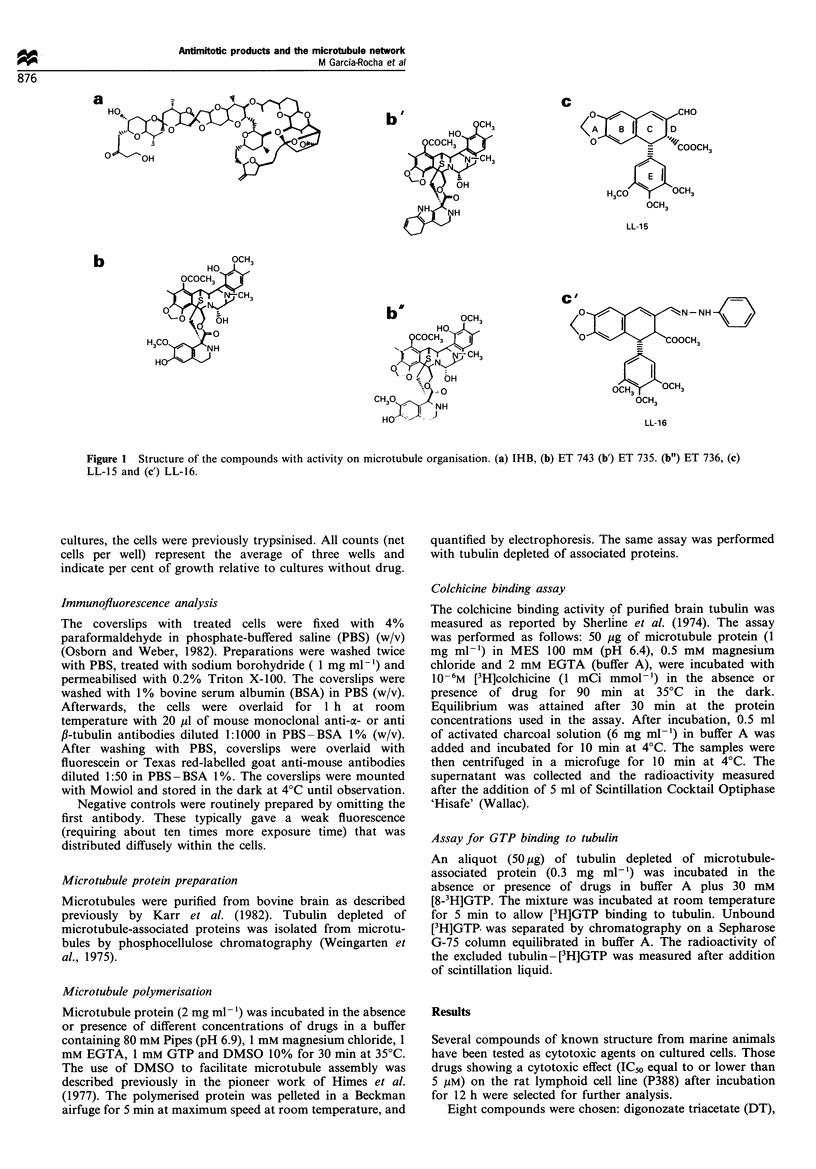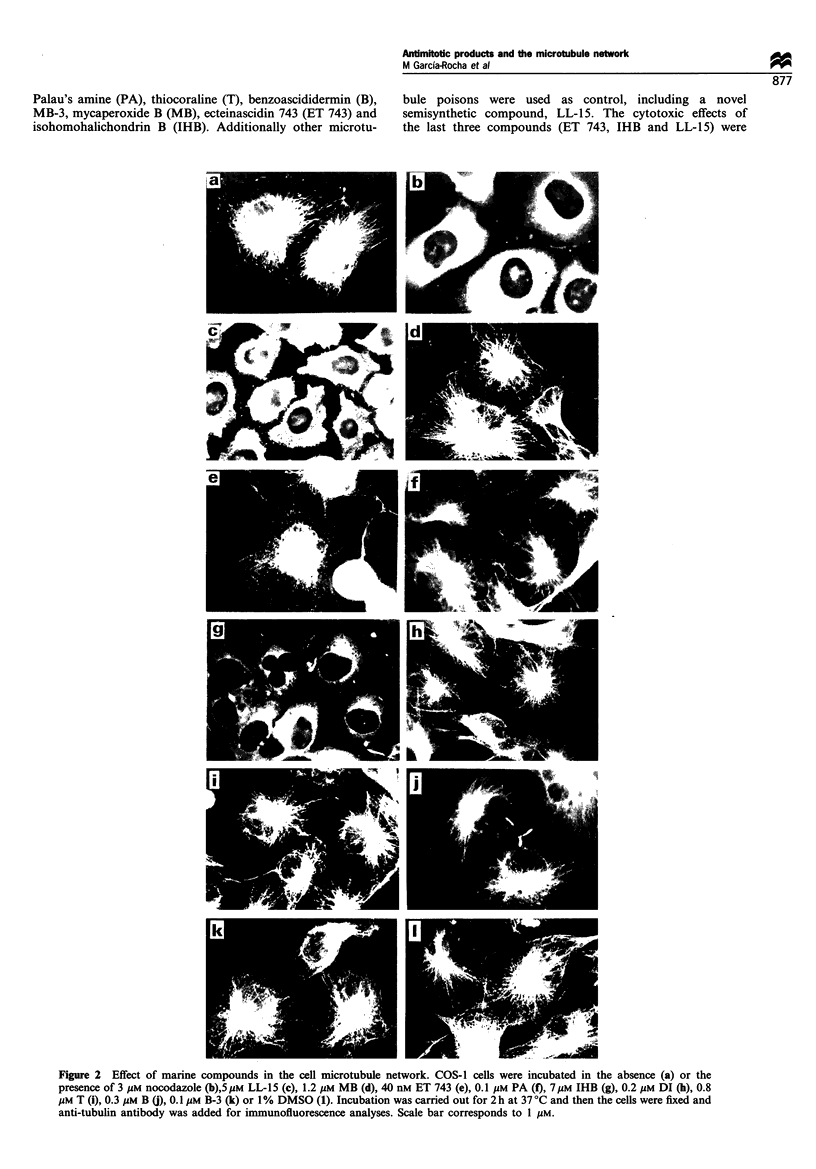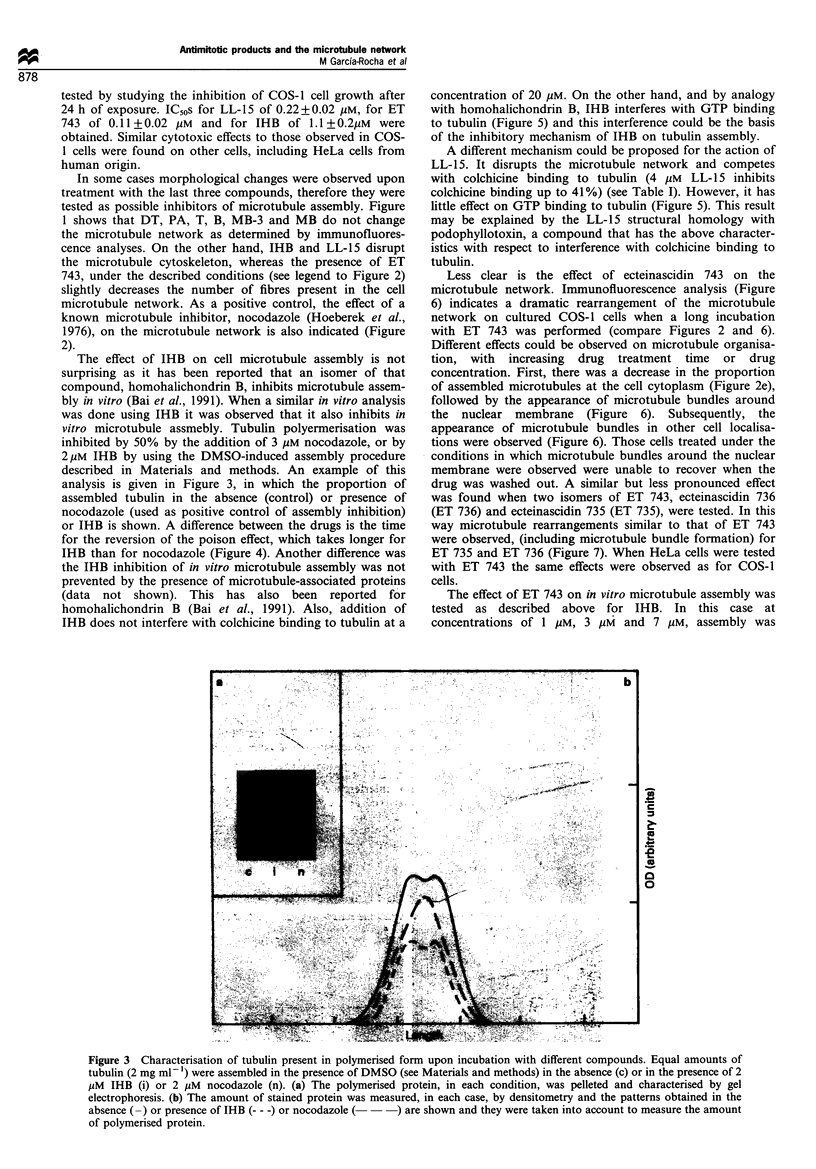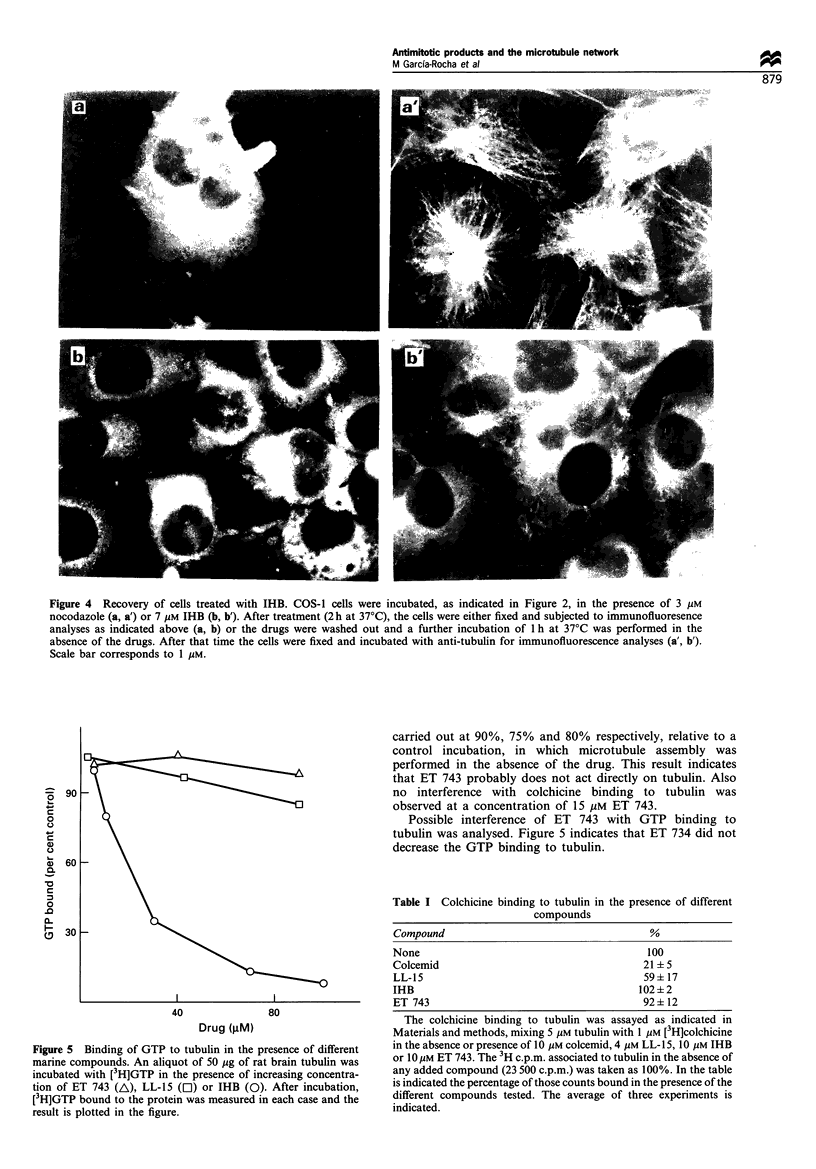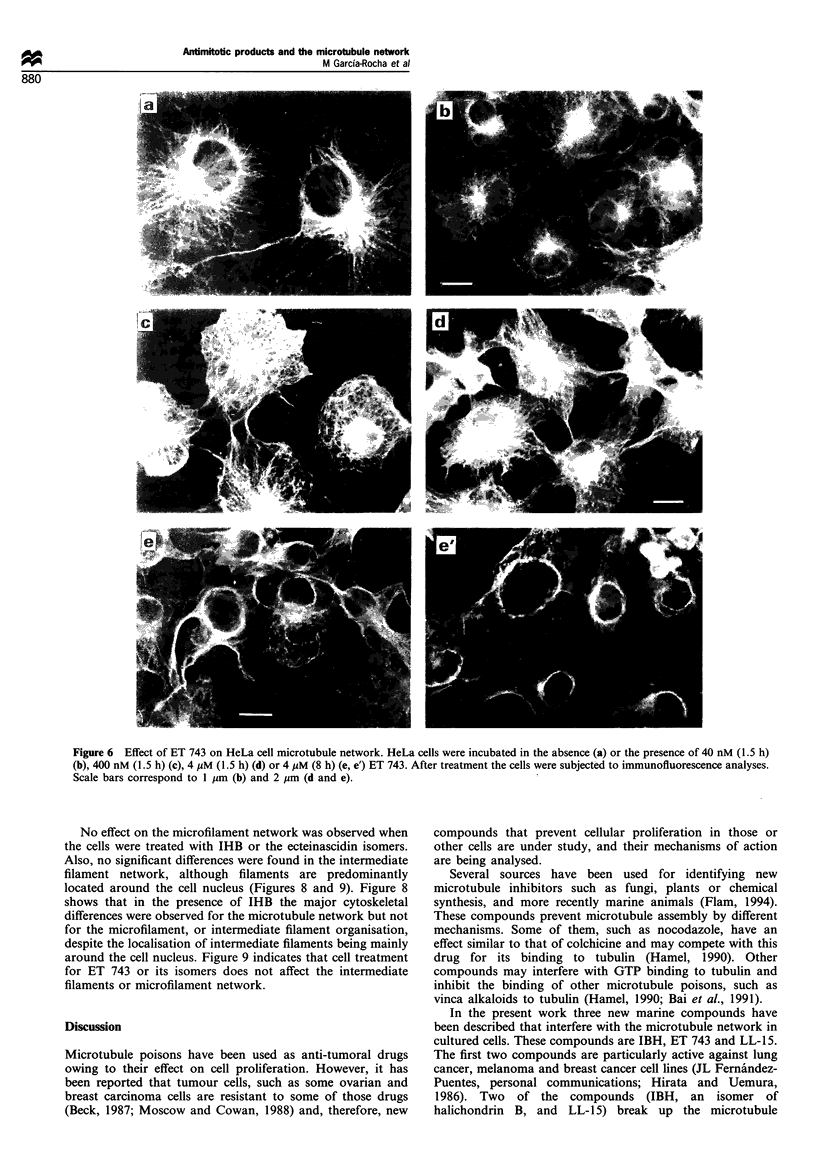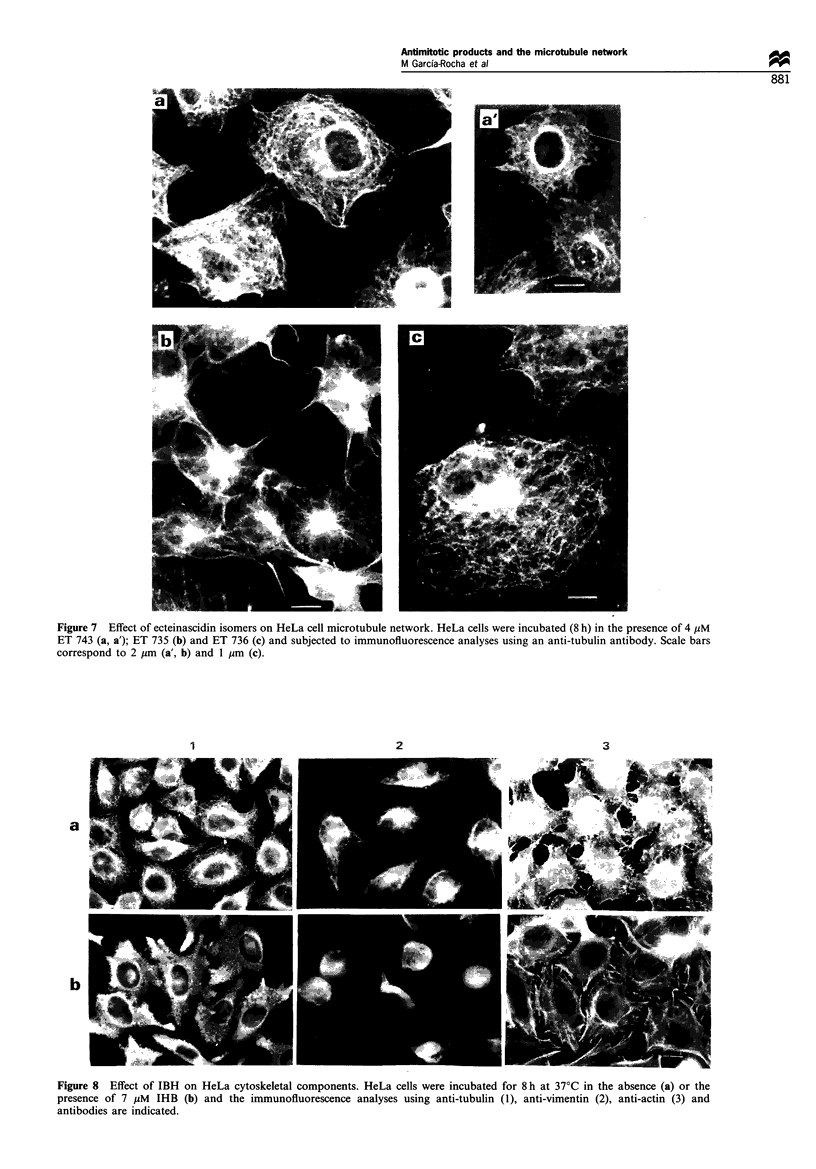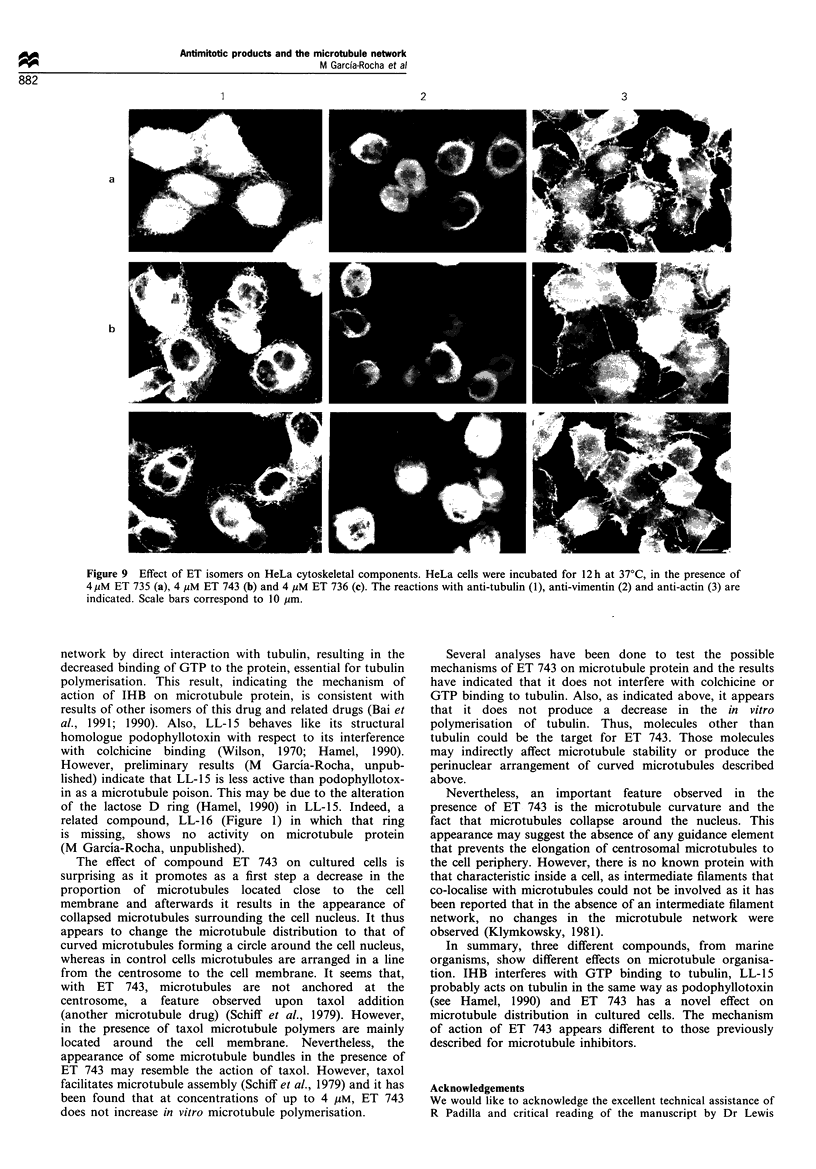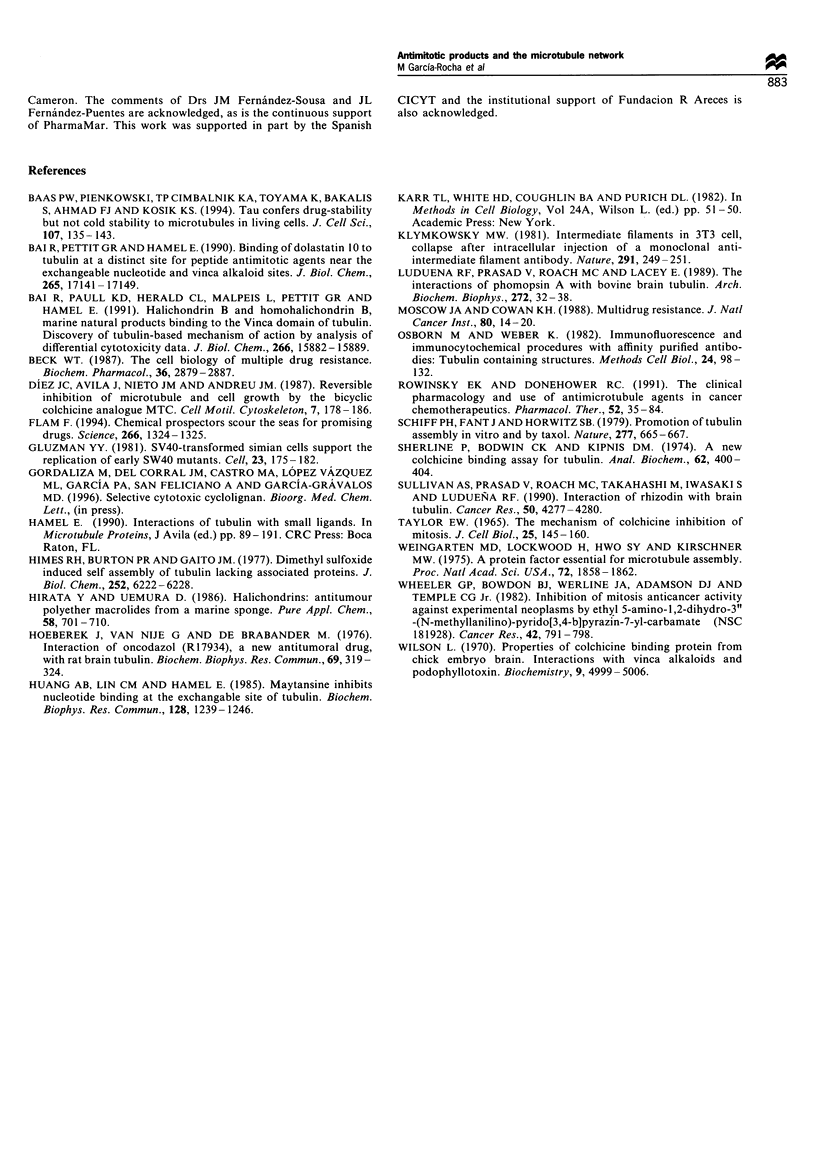# Characterisation of antimitotic products from marine organisms that disorganise the microtubule network: ecteinascidin 743, isohomohalichondrin-B and LL-15.

**DOI:** 10.1038/bjc.1996.176

**Published:** 1996-04

**Authors:** M. García-Rocha, M. D. García-Gravalos, J. Avila

**Affiliations:** Centro de Biología Molecular Severo Ochoa, Universidad Autónoma de Madrid, Spain.

## Abstract

**Images:**


British Journal of Cancer (1996) 73, 875-883
© 1996 Stockton Press All rights reserved 0007-0920/96 $12.00

### Characterisation of antimitotic products from marine organisms that disorganise the microtubule network: ecteinascidin 743, isohomohalichondrin-B and LL-15

M García-Rocha[1], MD García-Gravalos[2] and J Avila[1]

*Centro de Biología Molecular 'Severo Ochoa', Universidad Autónoma de Madrid, 28049-Cantoblanco, Madrid, Spain;*[2] *PharmaMar, c/De la Calera, 3, Tres Cantos 28760, Madrid, Spain.*

**Summary** The effect of selected marine compounds with anti-tumoral activity on the cell microtubule network was tested by immunofluorescence analyses, or by other *in vitro* analyses involving competition with colchicine or with GTP for tubulin binding and tubulin polymerisation, studies that were carried out in parallel with other microtubule poisons used as controls. Three compounds were found to disorganise the microtubule network: isohomohalichondrin B, LL-15 and ecsteinascidin 743. The first two compounds prevent microtubule assembly and GTP binding to tubulin. Ecteinascidin 743 disorganises the microtubule network but it does not seem to interact directly with tubulin.

**Keywords:** anti-tumoral compound; cytoskeletal poison; tubulin

Microtubules are tubulin polymers involved in different functions, including the determination of cell morphology, the regulation of intracellular organisation or to facilitate chromosome segregation, which takes place during the process of cell division. Thus, compounds that interfere with the mechanism of microtubule polymerisation could inhibit assembly of the mitotic spindle and consequently could prevent cell division. This characteristic has been taken into account when identifying those compounds as anti-tumoral agents. Different natural and synthetic compounds have been used as anti-tumoral drugs based on their ability to affect the microtubule polymerisation-depolymerisation process (Rowinsky and Donehower, 1991; Hamel, 1990; Diez *et al.*, 1987; Ludueña *et al.*, 1989; Bai *et al.*, 1991; Sullivan *et al.*, 1990; Huang *et al.*, 1985; Hoeberek *et al.*, 1976; Wheeler *et al.*, 1982; Schiff *et al.*, 1979). Different mechanisms have been postulated for the action of different microtubule poisons. Some of those compounds prevent microtubule polymerisation by binding to tubulin in a similar way to colchicine, the first compound described to affect microtubule assembly (Taylor, 1965). Other compounds interfere with the binding of GTP to tubulin (Bai *et al.*, 1991). Also, the presence of certain microtubule-associated proteins, which could be different in cells of different origin, could modulate the action of microtubule inhibitors (Boas *et al.*, 1994). As different tumour cells may be resistant to some of those microtubule poisons (Beck, 1987), a search for novel compounds from new sources should be carried out.

Natural antimicrotubular compounds mainly originate from plant or fungi, and they usually show their inhibitory effect in the range of $10^{-6}$M (Hamel, 1990). Synthetic or semi synthetic compounds have also been used. An example is nocodazol (Hoeberek *et al.*, 1976). In this work we tested a family of compounds from marine animals, as those animals have been indicated as a source for microtubule poisons (Bai *et al.*, 1991) that could interfere with cell proliferation (Flam, 1994). Additionally, a semisynthetic product, LL-15 (Gordaliza *et al.*, 1996), with a structure related to podophylotoxin, a microtubule poison (Hamel, 1990), was tested. Our results indicate that three of the tested compounds alter the cell microtubule network at a low concentration range.

Correspondence: J Avila
Received 12 July 1995; revised 6 November 1995; accepted 17 November 1995

These compounds are isohomohalichondrin B (from the sponge *Lissodendoryx* sp, with activity in lung cancer or melanoma cell lines), (Hirata and Uemura, 1986), ecteinascidin 743 (from the tunicate *Ecteinascidia turbinata*, also with activity in lung cancer and in breast cancer and melanoma cell lines) and the compound LL-15. In this study we characterised the antimitotic effect of these compounds.

### Materials and methods

### *Chemicals and antibodies*

Colcemid (demecolcine) and nocodazole were obtained from Sigma. Ring A-4-[$^3$H]colchicine and [8-$^3$H]GTP (specific activity 1 mCi $mmol^{-1}$ and 7.7 Ci $mmol^{-1}$ respectively) were from Amersham. Isohomohalichondrin B, ecteinascidin 743, LL-15 and the other marine compounds (digonozate triacetate, Palau's amine, thiocoraline, benzoascidermin, MB-3 and mycoperoxide B) were provided by PharmaMar. The structures of some of these compounds and those of some isomers are shown in Figure 1. Stock solutions of these drugs were made in dimethyl sulphoxide (DMSO) and stored at −20°C. Anti-tubulin, anti-actin and anti-vimentin monoclonal antibodies and fluorescein or Texas red-labelled goat anti-mouse immunoglobulins were from Amersham.

### *Cell culture*

COS-1 (Gluzman, 1981), HeLa or mouse P388 cells were grown in Dulbecco's modified Eagle medium supplemented with 5% fetal bovine serum. Cells were plated onto 10 × 10 mm coverslips in 24-well tissue culture plates at a density of 100 cells $mm^{-2}$ and cultured for 1 day. The cells were then treated with different concentrations of the drugs for 2 – 24 h. After the treatment, in selected cases, the culture medium containing the drug was removed and fresh medium was added to allow cell recovery.

### *Inhibition of growth*

COS-1, HeLa or P388 (lymphoid line) cells were seeded at $4 \times 10^3$ cells per well in 1 ml aliquots of culture medium, then incubated for 18 h. The medium was then replaced with culture medium containing the corresponding drug. All determinations were carried out in triplicate.

Cells were counted in a Coulter counter ZM at 6, 24, 48 and 72 h after drug addition. In the case of COS-1 and HeLa

**Figure 1** Structure of the compounds with activity on microtubule organisation. (**a**) IHB, (**b**) ET 743 (**b′**) ET 735. (**b″**) ET 736, (**c**) LL-15 and (**c′**) LL-16.

cultures, the cells were previously trypsinised. All counts (net cells per well) represent the average of three wells and indicate per cent of growth relative to cultures without drug.

*Immunofluorescence analysis*

The coverslips with treated cells were fixed with 4% paraformaldehyde in phosphate-buffered saline (PBS) (w/v) (Osborn and Weber, 1982). Preparations were washed twice with PBS, treated with sodium borohydride ( 1 mg $ml^{-1}$) and permeabilised with 0.2% Triton X-100. The coverslips were washed with 1% bovine serum albumin (BSA) in PBS (w/v). Afterwards, the cells were overlaid for 1 h at room temperature with 20 $\mu$l of mouse monoclonal anti-$\alpha$- or anti $\beta$-tubulin antibodies diluted 1:1000 in PBS–BSA 1% (w/v). After washing with PBS, coverslips were overlaid with fluorescein or Texas red-labelled goat anti-mouse antibodies diluted 1:50 in PBS–BSA 1%. The coverslips were mounted with Mowiol and stored in the dark at 4°C until observation.

Negative controls were routinely prepared by omitting the first antibody. These typically gave a weak fluorescence (requiring about ten times more exposure time) that was distributed diffusely within the cells.

*Microtubule protein preparation*

Microtubules were purified from bovine brain as described previously by Karr *et al.* (1982). Tubulin depleted of microtubule-associated proteins was isolated from microtubules by phosphocellulose chromatography (Weingarten *et al.*, 1975).

*Microtubule polymerisation*

Microtubule protein (2 mg $ml^{-1}$) was incubated in the absence or presence of different concentrations of drugs in a buffer containing 80 mM Pipes (pH 6.9), 1 mM magnesium chloride, 1 mM EGTA, 1 mM GTP and DMSO 10% for 30 min at 35°C. The use of DMSO to facilitate microtubule assembly was described previously in the pioneer work of Himes *et al.* (1977). The polymerised protein was pelleted in a Beckman airfuge for 5 min at maximum speed at room temperature, and quantified by electrophoresis. The same assay was performed with tubulin depleted of associated proteins.

*Colchicine binding assay*

The colchicine binding activity of purified brain tubulin was measured as reported by Sherline *et al.* (1974). The assay was performed as follows: 50 $\mu$g of microtubule protein (1 mg $ml^{-1}$) in MES 100 mM (pH 6.4), 0.5 mM magnesium chloride and 2 mM EGTA (buffer A), were incubated with $10^{-6}$M [$^3$H]colchicine (1 mCi $mmol^{-1}$) in the absence or presence of drug for 90 min at 35°C in the dark. Equilibrium was attained after 30 min at the protein concentrations used in the assay. After incubation, 0.5 ml of activated charcoal solution (6 mg $ml^{-1}$) in buffer A was added and incubated for 10 min at 4°C. The samples were then centrifuged in a microfuge for 10 min at 4°C. The supernatant was collected and the radioactivity measured after the addition of 5 ml of Scintillation Cocktail Optiphase 'Hisafe' (Wallac).

*Assay for GTP binding to tubulin*

An aliquot (50 $\mu$g) of tubulin depleted of microtubule-associated protein (0.3 mg $ml^{-1}$) was incubated in the absence or presence of drugs in buffer A plus 30 mM [8-$^3$H]GTP. The mixture was incubated at room temperature for 5 min to allow [$^3$H]GTP binding to tubulin. Unbound [$^3$H]GTP was separated by chromatography on a Sepharose G-75 column equilibrated in buffer A. The radioactivity of the excluded tubulin–[$^3$H]GTP was measured after addition of scintillation liquid.

### Results

Several compounds of known structure from marine animals have been tested as cytotoxic agents on cultured cells. Those drugs showing a cytotoxic effect ($IC_{50}$ equal to or lower than 5 $\mu$M) on the rat lymphoid cell line (P388) after incubation for 12 h were selected for further analysis.

Eight compounds were chosen: digonozate triacetate (DT),

Palau's amine (PA), thiocoraline (T), benzoascididermin (B), MB-3, mycaperoxide B (MB), ecteinascidin 743 (ET 743) and isohomohalichondrin B (IHB). Additionally other microtubule poisons were used as control, including a novel semisynthetic compound, LL-15. The cytotoxic effects of the last three compounds (ET 743, IHB and LL-15) were

**Figure 2** Effect of marine compounds in the cell microtubule network. COS-1 cells were incubated in the absence (**a**) or the presence of 3 $\mu$M nocodazole (**b**),5 $\mu$M LL-15 (**c**), 1.2 $\mu$M MB (**d**), 40 nM ET 743 (**e**), 0.1 $\mu$M PA (**f**), 7 $\mu$M IHB (**g**), 0.2 $\mu$M DI (**h**), 0.8 $\mu$M T (**i**), 0.3 $\mu$M B (**j**), 0.1 $\mu$M B-3 (**k**) or 1% DMSO (**l**). Incubation was carried out for 2 h at 37°C and then the cells were fixed and anti-tubulin antibody was added for immunofluorescence analyses. Scale bar corresponds to 1 $\mu$M.

tested by studying the inhibition of COS-1 cell growth after 24 h of exposure. $IC_{50}$s for LL-15 of 0.22±0.02 μM, for ET 743 of 0.11±0.02 μM and for IHB of 1.1±0.2μM were obtained. Similar cytotoxic effects to those observed in COS-1 cells were found on other cells, including HeLa cells from human origin.

In some cases morphological changes were observed upon treatment with the last three compounds, therefore they were tested as possible inhibitors of microtubule assembly. Figure 1 shows that DT, PA, T, B, MB-3 and MB do not change the microtubule network as determined by immunofluorescence analyses. On the other hand, IHB and LL-15 disrupt the microtubule cytoskeleton, whereas the presence of ET 743, under the described conditions (see legend to Figure 2) slightly decreases the number of fibres present in the cell microtubule network. As a positive control, the effect of a known microtubule inhibitor, nocodazole (Hoeberek *et al*., 1976), on the microtubule network is also indicated (Figure 2).

The effect of IHB on cell microtubule assembly is not surprising as it has been reported that an isomer of that compound, homohalichondrin B, inhibits microtubule assembly *in vitro* (Bai *et al*., 1991). When a similar *in vitro* analysis was done using IHB it was observed that it also inhibits *in vitro* microtubule assmebly. Tubulin polyermerisation was inhibited by 50% by the addition of 3 μM nocodazole, or by 2 μM IHB by using the DMSO-induced assembly procedure described in Materials and methods. An example of this analysis is given in Figure 3, in which the proportion of assembled tubulin in the absence (control) or presence of nocodazole (used as positive control of assembly inhibition) or IHB is shown. A difference between the drugs is the time for the reversion of the poison effect, which takes longer for IHB than for nocodazole (Figure 4). Another difference was the IHB inhibition of *in vitro* microtubule assembly was not prevented by the presence of microtubule-associated proteins (data not shown). This has also been reported for homohalichondrin B (Bai *et al*., 1991). Also, addition of IHB does not interfere with colchicine binding to tubulin at a concentration of 20 μM. On the other hand, and by analogy with homohalichondrin B, IHB interferes with GTP binding to tubulin (Figure 5) and this interference could be the basis of the inhibitory mechanism of IHB on tubulin assembly.

A different mechanism could be proposed for the action of LL-15. It disrupts the microtubule network and competes with colchicine binding to tubulin (4 μM LL-15 inhibits colchicine binding up to 41%) (see Table I). However, it has little effect on GTP binding to tubulin (Figure 5). This result may be explained by the LL-15 structural homology with podophyllotoxin, a compound that has the above characteristics with respect to interference with colchicine binding to tubulin.

Less clear is the effect of ecteinascidin 743 on the microtubule network. Immunofluorescence analysis (Figure 6) indicates a dramatic rearrangement of the microtubule network on cultured COS-1 cells when a long incubation with ET 743 was performed (compare Figures 2 and 6). Different effects could be observed on microtubule organisation, with increasing drug treatment time or drug concentration. First, there was a decrease in the proportion of assembled microtubules at the cell cytoplasm (Figure 2e), followed by the appearance of microtubule bundles around the nuclear membrane (Figure 6). Subsequently, the appearance of microtubule bundles in other cell localisations were observed (Figure 6). Those cells treated under the conditions in which microtubule bundles around the nuclear membrane were observed were unable to recover when the drug was washed out. A similar but less pronounced effect was found when two isomers of ET 743, ecteinascidin 736 (ET 736) and ecteinascidin 735 (ET 735), were tested. In this way microtubule rearrangements similar to that of ET 743 were observed, (including microtubule bundle formation) for ET 735 and ET 736 (Figure 7). When HeLa cells were tested with ET 743 the same effects were observed as for COS-1 cells.

The effect of ET 743 on *in vitro* microtubule assembly was tested as described above for IHB. In this case at concentrations of 1 μM, 3 μM and 7 μM, assembly was

**Figure 3** Characterisation of tubulin present in polymerised form upon incubation with different compounds. Equal amounts of tubulin (2 mg $ml^{-1}$) were assembled in the presence of DMSO (see Materials and methods) in the absence (c) or in the presence of 2 μM IHB (i) or 2 μM nocodazole (n). (**a**) The polymerised protein, in each condition, was pelleted and characterised by gel electrophoresis. (**b**) The amount of stained protein was measured, in each case, by densitometry and the patterns obtained in the absence (–) or presence of IHB (- - -) or nocodazole (— — —) are shown and they were taken into account to measure the amount of polymerised protein.

**Figure 4** Recovery of cells treated with IHB. COS-1 cells were incubated, as indicated in Figure 2, in the presence of 3 $\mu$M nocodazole (**a**, **a'**) or 7 $\mu$M IHB (**b**, **b'**). After treatment (2 h at 37°C), the cells were either fixed and subjected to immunofluoresence analyses as indicated above (**a**, **b**) or the drugs were washed out and a further incubation of 1 h at 37°C was performed in the absence of the drugs. After that time the cells were fixed and incubated with anti-tubulin for immunofluorescence analyses (**a'**, **b'**). Scale bar corresponds to 1 $\mu$M.

**Figure 5** Binding of GTP to tubulin in the presence of different marine compounds. An aliquot of 50 $\mu$g of rat brain tubulin was incubated with [$^3$H]GTP in the presence of increasing concentration of ET 743 (△), LL-15 (□) or IHB (○). After incubation, [$^3$H]GTP bound to the protein was measured in each case and the result is plotted in the figure.

carried out at 90%, 75% and 80% respectively, relative to a control incubation, in which microtubule assembly was performed in the absence of the drug. This result indicates that ET 743 probably does not act directly on tubulin. Also no interference with colchicine binding to tubulin was observed at a concentration of 15 $\mu$M ET 743.

Possible interference of ET 743 with GTP binding to tubulin was analysed. Figure 5 indicates that ET 734 did not decrease the GTP binding to tubulin.

**Table I** Colchicine binding to tubulin in the presence of different compounds

| *Compound* | % |
|---|---|
| None | 100 |
| Colcemid | 21 ± 5 |
| LL-15 | 59 ± 17 |
| IHB | 102 ± 2 |
| ET 743 | 92 ± 12 |

The colchicine binding to tubulin was assayed as indicated in Materials and methods, mixing 5 $\mu$M tubulin with 1 $\mu$M [$^3$H]colchicine in the absence or presence of 10 $\mu$M colcemid, 4 $\mu$M LL-15, 10 $\mu$M IHB or 10 $\mu$M ET 743. The $^3$H c.p.m. associated to tubulin in the absence of any added compound (23 500 c.p.m.) was taken as 100%. In the table is indicated the percentage of those counts bound in the presence of the different compounds tested. The average of three experiments is indicated.

**Figure 6** Effect of ET 743 on HeLa cell microtubule network. HeLa cells were incubated in the absence (**a**) or the presence of 40 nM (1.5 h) (**b**), 400 nM (1.5 h) (**c**), 4 $\mu$M (1.5 h) (**d**) or 4 $\mu$M (8 h) (**e**, **e'**) ET 743. After treatment the cells were subjected to immunofluorescence analyses. Scale bars correspond to 1 $\mu$m (**b**) and 2 $\mu$m (**d** and **e**).

No effect on the microfilament network was observed when the cells were treated with IHB or the ecteinascidin isomers. Also, no significant differences were found in the intermediate filament network, although filaments are predominantly located around the cell nucleus (Figures 8 and 9). Figure 8 shows that in the presence of IHB the major cytoskeletal differences were observed for the microtubule network but not for the microfilament, or intermediate filament organisation, despite the localisation of intermediate filaments being mainly around the cell nucleus. Figure 9 indicates that cell treatment for ET 743 or its isomers does not affect the intermediate filaments or microfilament network.

### Discussion

Microtubule poisons have been used as anti-tumoral drugs owing to their effect on cell proliferation. However, it has been reported that tumour cells, such as some ovarian and breast carcinoma cells are resistant to some of those drugs (Beck, 1987; Moscow and Cowan, 1988) and, therefore, new compounds that prevent cellular proliferation in those or other cells are under study, and their mechanisms of action are being analysed.

Several sources have been used for identifying new microtubule inhibitors such as fungi, plants or chemical synthesis, and more recently marine animals (Flam, 1994). These compounds prevent microtubule assembly by different mechanisms. Some of them, such as nocodazole, have an effect similar to that of colchicine and may compete with this drug for its binding to tubulin (Hamel, 1990). Other compounds may interfere with GTP binding to tubulin and inhibit the binding of other microtubule poisons, such as vinca alkaloids to tubulin (Hamel, 1990; Bai *et al.*, 1991).

In the present work three new marine compounds have been described that interfere with the microtubule network in cultured cells. These compounds are IBH, ET 743 and LL-15. The first two compounds are particularly active against lung cancer, melanoma and breast cancer cell lines (JL Fernández-Puentes, personal communications; Hirata and Uemura, 1986). Two of the compounds (IBH, an isomer of halichondrin B, and LL-15) break up the microtubule

**Figure 7** Effect of ecteinascidin isomers on HeLa cell microtubule network. HeLa cells were incubated (8 h) in the presence of 4 $\mu$M ET 743 (**a**, **a'**); ET 735 (**b**) and ET 736 (**c**) and subjected to immunofluorescence analyses using an anti-tubulin antibody. Scale bars correspond to 2 $\mu$m (**a'**, **b**) and 1 $\mu$m (**c**).

**Figure 8** Effect of IBH on HeLa cytoskeletal components. HeLa cells were incubated for 8 h at 37°C in the absence (**a**) or the presence of 7 $\mu$M IHB (**b**) and the immunofluorescence analyses using anti-tubulin (1), anti-vimentin (2), anti-actin (3) and antibodies are indicated.

**Figure 9** Effect of ET isomers on HeLa cytoskeletal components. HeLa cells were incubated for 12 h at 37°C, in the presence of 4 $\mu$M ET 735 (**a**), 4 $\mu$M ET 743 (**b**) and 4 $\mu$M ET 736 (**c**). The reactions with anti-tubulin (1), anti-vimentin (2) and anti-actin (3) are indicated. Scale bars correspond to 10 $\mu$m.

network by direct interaction with tubulin, resulting in the decreased binding of GTP to the protein, essential for tubulin polymerisation. This result, indicating the mechanism of action of IHB on microtubule protein, is consistent with results of other isomers of this drug and related drugs (Bai *et al.*, 1991; 1990). Also, LL-15 behaves like its structural homologue podophyllotoxin with respect to its interference with colchicine binding (Wilson, 1970; Hamel, 1990). However, preliminary results (M García-Rocha, unpublished) indicate that LL-15 is less active than podophyllotoxin as a microtubule poison. This may be due to the alteration of the lactose D ring (Hamel, 1990) in LL-15. Indeed, a related compound, LL-16 (Figure 1) in which that ring is missing, shows no activity on microtubule protein (M García-Rocha, unpublished).

The effect of compound ET 743 on cultured cells is surprising as it promotes as a first step a decrease in the proportion of microtubules located close to the cell membrane and afterwards it results in the appearance of collapsed microtubules surrounding the cell nucleus. It thus appears to change the microtubule distribution to that of curved microtubules forming a circle around the cell nucleus, whereas in control cells microtubules are arranged in a line from the centrosome to the cell membrane. It seems that, with ET 743, microtubules are not anchored at the centrosome, a feature observed upon taxol addition (another microtubule drug) (Schiff *et al.*, 1979). However, in the presence of taxol microtubule polymers are mainly located around the cell membrane. Nevertheless, the appearance of some microtubule bundles in the presence of ET 743 may resemble the action of taxol. However, taxol facilitates microtubule assembly (Schiff *et al.*, 1979) and it has been found that at concentrations of up to 4 $\mu$M, ET 743 does not increase *in vitro* microtubule polymerisation.

Several analyses have been done to test the possible mechanisms of ET 743 on microtubule protein and the results have indicated that it does not interfere with colchicine or GTP binding to tubulin. Also, as indicated above, it appears that it does not produce a decrease in the *in vitro* polymerisation of tubulin. Thus, molecules other than tubulin could be the target for ET 743. Those molecules may indirectly affect microtubule stability or produce the perinuclear arrangement of curved microtubules described above.

Nevertheless, an important feature observed in the presence of ET 743 is the microtubule curvature and the fact that microtubules collapse around the nucleus. This appearance may suggest the absence of any guidance element that prevents the elongation of centrosomal microtubules to the cell periphery. However, there is no known protein with that characteristic inside a cell, as intermediate filaments that co-localise with microtubules could not be involved as it has been reported that in the absence of an intermediate filament network, no changes in the microtubule network were observed (Klymkowsky, 1981).

In summary, three different compounds, from marine organisms, show different effects on microtubule organisation. IHB interferes with GTP binding to tubulin, LL-15 probably acts on tubulin in the same way as podophyllotoxin (see Hamel, 1990) and ET 743 has a novel effect on microtubule distribution in cultured cells. The mechanism of action of ET 743 appears different to those previously described for microtubule inhibitors.

**Acknowledgements**
We would like to acknowledge the excellent technical assistance of R Padilla and critical reading of the manuscript by Dr Lewis

Cameron. The comments of Drs JM Fernández-Sousa and JL Fernández-Puentes are acknowledged, as is the continuous support of PharmaMar. This work was supported in part by the Spanish CICYT and the institutional support of Fundacion R Areces is also acknowledged.

### References

BAAS PW, PIENKOWSKI, TP CIMBALNIK KA, TOYAMA K, BAKALIS S, AHMAD FJ AND KOSIK KS. (1994). Tau confers drug-stability but not cold stability to microtubules in living cells. *J. Cell Sci.*, **107**, 135–143.

BAI R, PETTIT GR AND HAMEL E. (1990). Binding of dolastatin 10 to tubulin at a distinct site for peptide antimitotic agents near the exchangeable nucleotide and vinca alkaloid sites. *J. Biol. Chem.*, **265**, 17141–17149.

BAI R, PAULL KD, HERALD CL, MALPEIS L, PETTIT GR AND HAMEL E. (1991). Halichondrin B and homohalichondrin B, marine natural products binding to the Vinca domain of tubulin. Discovery of tubulin-based mechanism of action by analysis of differential cytotoxicity data. *J. Biol. Chem.*, **266**, 15882–15889.

BECK WT. (1987). The cell biology of multiple drug resistance. *Biochem. Pharmacol.*, **36**, 2879–2887.

DÍEZ JC, AVILA J, NIETO JM AND ANDREU JM. (1987). Reversible inhibition of microtubule and cell growth by the bicyclic colchicine analogue MTC. *Cell Motil. Cytoskeleton*, **7**, 178–186.

FLAM F. (1994). Chemical prospectors scour the seas for promising drugs. *Science*, **266**, 1324–1325.

GLUZMAN YY. (1981). SV40-transformed simian cells support the replication of early SW40 mutants. *Cell*, **23**, 175–182.

GORDALIZA M, DEL CORRAL JM, CASTRO MA, LÓPEZ VÁZQUEZ ML, GARCÍA PA, SAN FELICIANO A AND GARCÍA-GRÁVALOS MD. (1996). Selective cytotoxic cyclolignan. *Bioorg. Med. Chem. Lett.*, (in press).

HAMEL E. (1990). Interactions of tubulin with small ligands. In *Microtubule Proteins*, J Avila (ed.) pp. 89–191. CRC Press: Boca Raton, FL.

HIMES RH, BURTON PR AND GAITO JM. (1977). Dimethyl sulfoxide induced self assembly of tubulin lacking associated proteins. *J. Biol. Chem.*, **252**, 6222–6228.

HIRATA Y AND UEMURA D. (1986). Halichondrins: antitumour polyether macrolides from a marine sponge. *Pure Appl. Chem.*, **58**, 701–710.

HOEBEREK J, VAN NIJE G AND DE BRABANDER M. (1976). Interaction of oncodazol (R17934), a new antitumoral drug, with rat brain tubulin. *Biochem. Biophys. Res. Commun.*, **69**, 319–324.

HUANG AB, LIN CM AND HAMEL E. (1985). Maytansine inhibits nucleotide binding at the exchangable site of tubulin. *Biochem. Biophys. Res. Commun.*, **128**, 1239–1246.

KARR TL, WHITE HD, COUGHLIN BA AND PURICH DL. (1982). In *Methods in Cell Biology*, Vol 24A, Wilson L. (ed.) pp. 51–50. Academic Press: New York.

KLYMKOWSKY MW. (1981). Intermediate filaments in 3T3 cell, collapse after intracellular injection of a monoclonal anti-intermediate filament antibody. *Nature*, **291**, 249–251.

LUDUENA RF, PRASAD V, ROACH MC AND LACEY E. (1989). The interactions of phomopsin A with bovine brain tubulin. *Arch. Biochem. Biophys.*, **272**, 32–38.

MOSCOW JA AND COWAN KH. (1988). Multidrug resistance. *J. Natl Cancer Inst.*, **80**, 14–20.

OSBORN M AND WEBER K. (1982). Immunofluorescence and immunocytochemical procedures with affinity purified antibodies: Tubulin containing structures. *Methods Cell Biol.*, **24**, 98–132.

ROWINSKY EK AND DONEHOWER RC. (1991). The clinical pharmacology and use of antimicrotubule agents in cancer chemotherapeutics. *Pharmacol. Ther.*, **52**, 35–84.

SCHIFF PH, FANT J AND HORWITZ SB. (1979). Promotion of tubulin assembly in vitro and by taxol. *Nature*, **277**, 665–667.

SHERLINE P, BODWIN CK AND KIPNIS DM. (1974). A new colchicine binding assay for tubulin. *Anal. Biochem.*, **62**, 400–404.

SULLIVAN AS, PRASAD V, ROACH MC, TAKAHASHI M, IWASAKI S AND LUDUEÑA RF. (1990). Interaction of rhizodin with brain tubulin. *Cancer Res.*, **50**, 4277–4280.

TAYLOR EW. (1965). The mechanism of colchicine inhibition of mitosis. *J. Cell Biol.*, **25**, 145–160.

WEINGARTEN MD, LOCKWOOD H, HWO SY AND KIRSCHNER MW. (1975). A protein factor essential for microtubule assembly. *Proc. Natl Acad. Sci. USA.*, **72**, 1858–1862.

WHEELER GP, BOWDON BJ, WERLINE JA, ADAMSON DJ AND TEMPLE CG Jr. (1982). Inhibition of mitosis anticancer activity against experimental neoplasms by ethyl 5-amino-1,2-dihydro-3"-(N-methyllanilino)-pyrido[3,4-b]pyrazin-7-yl-carbamate (NSC 181928). *Cancer Res.*, **42**, 791–798.

WILSON L. (1970). Properties of colchicine binding protein from chick embryo brain. Interactions with vinca alkaloids and podophyllotoxin. *Biochemistry*, **9**, 4999–5006.